# Dielectric properties and carbothermic reduction of zinc oxide and zinc ferrite by microwave heating

**DOI:** 10.1098/rsos.170710

**Published:** 2017-09-20

**Authors:** Mamdouh Omran, Timo Fabritius, Eetu-Pekka Heikkinen, Guo Chen

**Affiliations:** 1 Process Metallurgy Research Group, Faculty of Technology, University of Oulu, Oulu, Finland; 2 Mineral Processing and Agglomeration Laboratory, Central Metallurgical Research and Development Institute, Cairo, Egypt; 3 Key Laboratory of Unconventional Metallurgy, Kunming University of Science and Technology, Kunming, People's Republic of China

**Keywords:** zinc oxide, zinc ferrite, dielectric properties, microwave heating, carbothermic reduction

## Abstract

This paper aims to study the dielectric properties and carbothermic reduction of zinc oxide (zincite, ZnO) and zinc ferrite (franklinite, ZnFe_2_O_4_) by microwave heating. To achieve this aim, the dielectric properties were measured with an open-ended coaxial method to understand the behaviour of the samples under microwave irradiation. The effects of microwave power, duration time and sample mass on the heating rate, and the effects of the stoichiometric amount of graphite on the reduction of ZnO and decomposition of ZnFe_2_O_4_ were investigated. The results show that ZnFe_2_O_4_ has significantly higher dielectric properties compared to ZnO. Generally, for both samples, the dielectric values at room temperature were quite low, indicating that both ZnO and ZnFe_2_O_4_ are poor microwave absorbers. It was found that the temperatures have a more significant effect on the imaginary permittivities than on the real permittivities. The heating rate showed that the sample temperature increased with increase in microwave power and sample mass. Using 700 W of microwave power and two times the stoichiometric amount of graphite, almost complete reduction of ZnO was achieved in 12 min, while ZnFe_2_O_4_ completely decomposed to zincite and wustite in 3 min.

## 1. Introduction

Recently, there has been a growing interest in microwave energy as an alternative heating source. Microwave energy is a new promising technology which can be applied in the processing of primary and secondary raw materials. Microwave energy is a non-ionizing form of electromagnetic radiation with frequencies in the range of 300 MHz to 300 GHz [1,2]. Microwave heating is fundamentally different from conventional heating because microwaves take the form of electromagnetic energy and can penetrate deep into the sample. This allows sample heating to be initiated volumetrically, as opposed to conventional thermal processing which heats the sample from the outside inwards via standard heat transfer mechanisms, i.e. through convection, conduction and radiation [3]. The microwave heating rate is high and this results in the shortening of the heating time [3,4]. Therefore, the disadvantages of conventional heating methods such as large temperature gradient, long processing time and high energy consumption can be avoided with microwave heating [3,5].

A growing interest in microwave applications has emerged in recent years. These include microwave-assisted ore grinding, microwave-assisted carbothermic reduction of metal oxides, microwave-assisted drying and anhydration, microwave-assisted mineral leaching, microwave-assisted roasting and smelting of sulfide concentrate, and microwave-assisted waste management [6,7].

Wu *et al.* [8] investigated the effect of microwave irradiation on the phase transformation and the magnetic properties of limonite ore under reductive condition. He concluded that the microwave roasting process in the presence of alkali lignin could be a promising approach to effective use of limonite ore resources. Chen *et al.* [9] studied the decomposition of manganese ore using microwave heating. It was concluded that microwave heating can be applied effectively and efficiently to the calcination processes of the manganese ore.

There is a paucity of information on the microwave heating behaviour of zinc oxide (ZnO) and zinc ferrite (ZnFe_2_O_4_). A few papers have been published and most of this literature has focused on the study of the reduction behaviour of ZnO and ZnFe_2_O_4_ under microwave irradiation. Wang *et al*. [10] studied the thermodynamics of carbothermal reduction of ZnFe_2_O_4_ by microwave heating. He indicated the decomposition of ZnFe_2_O_4_ to ZnO and Fe_3_O_4_/FeO phases. Saidi & Azari [11] studied the carbothermic reduction of ZnO by microwaves, and concluded that increasing the microwave power and size of the sample could also increase the reduction rate. As a whole, microwave heating behaviour of ZnO and ZnFe_2_O_4_ has not been discussed widely in the previous investigations.

The aim of the present work is to give a comprehensive study on the microwave heating behaviour of ZnO and ZnFe_2_O_4_. Thus, this study examines the dielectric values of ZnO and ZnFe_2_O_4_, in order to understand their heating behaviour under microwave irradiation. The influence of temperature on the real and imaginary permittivities of ZnO and ZnFe_2_O_4_ at 1064 and 2423 MHz frequencies were measured. The heating characteristics and reduction behaviour of ZnO and ZnFe_2_O_4_ under microwave heating were investigated.

## 2. Microwave heating mechanism

The behaviour of material in a microwave field is determined by the magnetic (complex permeability, μ) and the dielectric properties (complex permittivity, ε) of the material [4]. For a material which does not have significant magnetic properties, complex permittivity is the major property that defines the microwave absorption of a material [12].

Complex permittivity ε is expressed as

2.1 $$\varepsilon =\varepsilon^{\prime }-j\varepsilon^{\prime \prime },$$

where $\varepsilon^{\prime }$ is the real part of the complex permittivity, $\varepsilon^{\prime \prime }$ is the imaginary part of the complex permittivity and *j* = (−1)^1/2^.

The real permittivity ($\varepsilon^{\prime }$) and the imaginary permittivity ($\varepsilon^{\prime \prime }$) are conventionally termed dielectric constant and dielectric loss factor, respectively. The dielectric constant expresses the ability of the material to absorb electromagnetic radiation within its structure, whereas the loss factor represents the ability of a material to dissipate the adsorbed radiation into heat [4,13]. The heating of a material depends greatly on the ratio of the loss factor of the material to the dielectric constant. Materials with a high loss factor are easily heated by microwave energy [14].

The loss tangent (tan *δ*_d_) is an important factor which provides an indication of how well a material dissipates stored energy into heat [13]. The loss tangent (tan *δ*_d_) is expressed as

2.2 tan δd=ε′′ε′.

Microwave heating uses the ability of some materials to absorb electromagnetic energy in the microwave spectral range and transform it into heat [14]. This heat spreads through the volume through conduction [15].

## 3. Material and methods

In this study, the reagent-grade chemicals used were ZnO (Alfa Aesar, 99.0%) and zinc iron oxide (Alfa Aesar, 99.0%). Synthetic graphite (greater than 99% purity, Alfa Aesar) was used as the reducing agent. Table 1 presents the properties of these materials.

**Table 1.** Properties of reagent-grade chemical materials.

phase	purity	molecular formula	formula weight	physical form	density
zinc oxide	99.0%	ZnO	81.37	−325 mesh powder	5.606 (5.16 g cm^−3^)
zinc iron oxide	99.0%	ZnFe_2_O_4_	241.06	−325 mesh powder	4.438 g cm^−3^
graphite	99.99%	C	12	−325 mesh powder	

## 4. Experimental

### 4.1. Dielectric measurement

The densities of the samples (ZnO and ZnFe_2_O_4_) were measured by the Archimedes method using deionized water and a 10.115 cm^3^ pycnometer (Gay-Lussac BlauBrand®, Brand GmbH + Co KG, Germany). The dielectric properties of the samples were measured with an open-ended coaxial probe method at 2.42 GHz and 1064 MHz frequencies. Details of the procedure are described in the paper [16]. It should be noted that the dielectric values of powder particles can differ from the values of solid material. This is due to electromagnetic dispersion and scattering effects caused by small particles [17].

First, the dielectric characterization of the samples was performed at room temperature (23.5°C) and humidity (45% RH). The effect of temperature on the real and imaginary permittivities of ZnO and ZnFe_2_O_4_ at 2423 and 1064 MHz frequencies were measured. In the experiments, sample powder is sealed in a resonant cavity (inner diameter of 80 mm, length of 100 mm) made of stainless steel and heated by an electric furnace placed inside the holder cavity. At least two duplicate measurements were carried out for each sample.

### 4.2. Microwave experimental set-up

**Figure 1.**
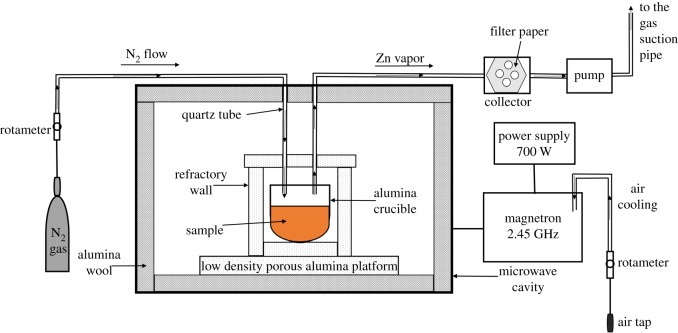
Schematic diagram of the microwave experimental set-up.

Diagrams of the microwave experimental set-up are shown in figure 1. A 2.45 GHz microwave oven with 700 W maximum output power was used in the experiments. Powder samples were placed in a microwave transparent alumina crucible, which was placed in a microwave transparent alumina refractory block and positioned in the centre of the microwave furnace on a low-density porous alumina platform.

The microwave absorption ability of samples at different microwave power intensities (500, 600 and 700 W) and processing times were measured. The temperature of the test sample was measured using a stainless steel-sheathed, K type thermocouple.

**Figure 2.**
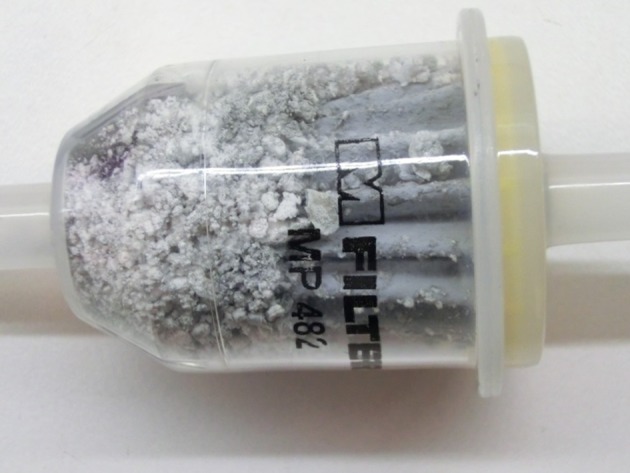
Evaporated zinc collected in filter paper.

The samples were well mixed with synthetic graphite (99.99% purity) at the different stoichiometric amounts. Nitrogen gas was used to maintain the reducing condition inside the microwave oven; an N_2_ flow rate of 5 ml min^−1^ was applied. The vapours from the crucible were pulled by the pump and trapped in the collector (figure 2). A filter paper was used inside the collector to catch the condensed particles. Compressed air was used to cool the magnetron during experiments.

### 4.3. Carbothermic reduction

ZnO, ZnFe_2_O_4_ and graphite were dried at 115°C overnight. Then, ZnO and ZnFe_2_O_4_ were well mixed with synthetic graphite (99.99% purity) at different stoichiometric amounts. After the experiments, the sample was weighed, ground and analysed to determine the degree of reduction.

The degree of ZnO reduction was defined as the amount of metal recovered from the corresponding metal oxide and was calculated as follows:

4.1 $$R=\left(\frac{m_{m}}{m_{0}}\right)\times 100\%.$$

Here *m*_m_ is the mass of ZnO in metallic form after reduction and *m*_0_ is the original mass of ZnO in oxide form [18].

The rate of ZnFe_2_O_4_ decomposition with time was calculated with X-ray diffraction (XRD) analyses; the reduced samples were weighed, ground and analysed by XRD to investigate the decomposition of ZnFe_2_O_4_.

The stoichiometric amount of carbon required to completely reduce ZnO or ZnFe_2_O_4_ into elemental zinc may be calculated based on the reduction reactions presented in equations (4.2)–(4.7). It should be noted that the required amount of carbon depends heavily on whether carbon is assumed to react into carbon monoxide (CO) or carbon dioxide (CO_2_) and whether iron included in ZnFe_2_O_4_ is assumed to also be reduced or not. The reduction reactions using different assumptions are presented in equations (4.2)–(4.3) for the reduction of ZnO and in equations (4.4)–(4.7) for the reduction of ZnFe_2_O_4_.

4.2 $$\begin{array}{rl} & \text{ZnO}+\text{C}=\text{Zn}+\text{CO}\;(\text{C reacts into CO}), \end{array}$$

4.3 $$\begin{array}{rl} & 2\text{ZnO}+\text{C}=2\text{Zn}+{\text{CO}}_{2}\;({\text{C reacts into CO}}_{2}), \end{array}$$

4.4 $$\begin{array}{rl} & {\text{ZnFe}}_{2}{\text{O}}_{4}+\text{C}=\text{Zn}+{\text{Fe}}_{2}{\text{O}}_{3}+\text{CO}\;(\text{C reacts into CO, iron is not reduced}), \end{array}$$

4.5 $$\begin{array}{rl} & 2{\text{ZnFe}}_{2}{\text{O}}_{4}+\text{C}=2\text{Zn}+2{\text{Fe}}_{2}{\text{O}}_{3}+{\text{CO}}_{2}\;({\text{C reacts into CO}}_{2},\text{iron is not reduced}), \end{array}$$

4.6 $$\begin{array}{rl} & {\text{ZnFe}}_{2}{\text{O}}_{4}+4\text{C}=\text{Zn}+2\text{Fe}+4\text{CO}\;(\text{C reacts into CO, iron is reduced}) \end{array}$$

4.7 $$\begin{array}{rl} \text{and}\;\; & {\text{ZnFe}}_{2}{\text{O}}_{4}+2\text{C}=\text{Zn}+2\text{Fe}+2{\text{CO}}_{2}\;({\text{C reacts into CO}}_{2},\text{iron is reduced}). \end{array}$$

### 4.4. Thermoanalyses

Differential scanning calorimetry (DSC) and thermogravimetry (TG) were used to determine the thermal behaviour of ZnO and ZnFe_2_O_4_. TG gives information about the temperature-dependent mass loss of the sample, while DSC detected the thermal reactions. The DSC–TG was performed using a Netzsch STA409 PC Luxx under N_2_ atmosphere. Approximately 23.84 mg of sample was placed in a platinum crucible on a pan of the microbalance at a heating rate of 10°C min^−1^. The temperature range was 20–1400°C.

### 4.5. X-ray diffraction

The phase transformation of ZnFe_2_O_4_ after microwave heating for different times was measured using Rigaku SmartLab 9 kW. The XRD analyses were done using operating conditions with an accelerating voltage of 40 kV and a current of 40 mA with a cobalt tube and a graphite monochromator. The measuring range was from 4° to 90° 2*θ* using a step size of 0.02° 2*θ* and a step time of 1 s per step.

### 4.6. Scanning electron microscope

The microstructure and microanalyses of phase transformation of ZnFe_2_O_4_ during microwave heating were investigated by combined scanning electron microscopy (SEM)–energy-dispersive X-ray spectroscopy (EDS) using a Zeiss ULTRA plus field emission scanning electron microscope, which was attached to an EDS unit for chemical analysis. To investigate the morphology of the phases, samples were investigated without polishing their surfaces. Small amounts of the samples were dispersed on the sample holder and coated with carbon.

## 5. Results and discussion

### 5.1. Thermoanalyses

The behaviour of ZnO and ZnFe_2_O_4_ as a function of temperature in the absence of any reducing agent was firstly studied. Then, the samples were mixed with two times the stoichiometric amount of carbon required to completely reduce ZnO and ZnFe_2_O_4_. (Carbon is assumed to react into carbon monoxide (CO); see §4.3.)

#### 5.1.1. Zinc oxide

**Figure 3.**
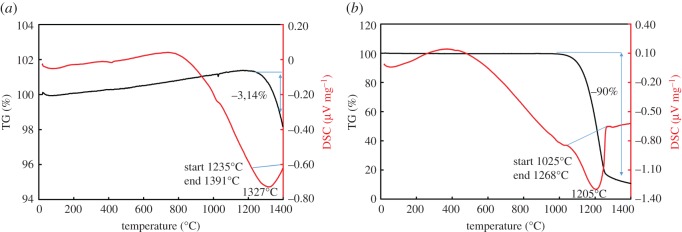
TG–DSC profile of ZnO. (*a*) Raw sample; (*b*) with reducing agent. (The *y*-axes scale in (*a*) and (*b*) figures are different).

The thermal behaviour of ZnO as a function of temperature is shown in figure 3*a*. The TG–DSC curve did not confirm thermal reaction or mass loss at temperatures below 1200°C (figure 3*a*). Therefore, we can conclude that there is no phase change. At high temperatures, 1235–1391°C, a deep valley in the DSC plot was due to the sintering of the sample. Figure 5*a* shows that the ZnO is sintering at high temperature, while the XRD pattern indicated that there is no phase change in the sample.

The behaviour of the ZnO under reducing conditions is shown in figure 3*b*, and this can be compared with the results in the absence of any reducing agent (figure 3*a*). The reduction of ZnO begins at temperature 1025°C; when the temperature reaches 1265°C, almost 100% of Zn is reduced and vaporized [18] (figure 3*b*). The TG curve shows a mass loss of about 90% due to the complete evaporation of zinc, and graphite gasification (that causes the formation of the CO/CO_2_ atmosphere), while the remaining mass is attributed to graphite. Figure 5*b* shows that some graphite is remaining in the crucible after the complete reduction of ZnO.

The reaction of ZnO with carbon can be expressed as [18,19]

5.1 $${\text{ZnO}}_{\left(s\right)}+{\text{C}}_{\left(s\right)}={\mathrm{Zn}}_{\left(g\right)}+{\mathrm{CO}}_{\left(g\right)}.$$

This reaction is a combination of the following reactions:

5.2 $${\text{ZnO}}_{\left(s\right)}+{\mathrm{CO}}_{\left(g\right)}={\text{Zn}}_{\left(g\right)}+{\text{CO}}_{2(g)}$$

and

5.3 $${\text{CO}}_{2(g)}+{\text{C}}_{\left(s\right)}=2{\text{CO}}_{\left(g\right)}.$$

The reduction mechanism can be described as follows: (i) carbon monoxide is produced by Boudouard reactions (reaction (5.3)); (ii) the ZnO is reduced by carbon monoxide to zinc vapour (reaction (5.2)) and (iii) carbon dioxide takes part in the reaction (5.3) again.

#### 5.1.2. Zinc ferrite

**Figure 4.**
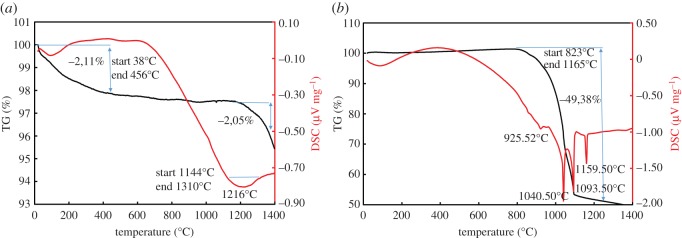
TG–DSC profile of ZnFe_2_O_4_. (*a*) Raw sample; (*b*) with reducing agent. (The *y*-axes scale in (*a*) and (*b*) figures are different).

**Figure 5.**
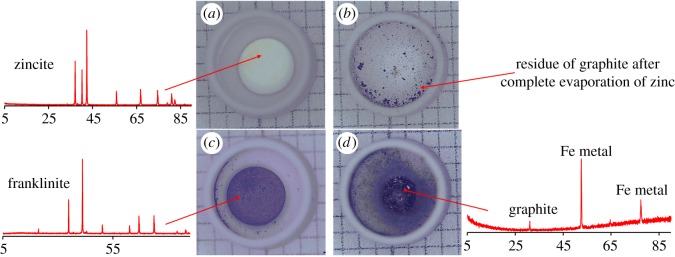
Experiment crucible after TG-DSC. (*a*) Raw ZnO; (*b*) ZnO with reducing agent; (*c*) Raw ZnFe_2_O_4_; (*d*) ZnFe_2_O_4_ with reducing agent.

The thermal behaviour of ZnFe_2_O_4_ in the absence of a reducing agent is shown in figure 4*a*. TG shows a mass loss of about 2% from 38°C to 456°C. This mass loss is attributed to the evaporation of physically adsorbed water. At high temperature, 1144–1310°C, a deep valley in the DSC plot is due to the sintering of the sample. Figure 5*c* shows the ZnFe_2_O_4_ is sintering at 1216°C, and XRD analysis indicated that there is no phase transformation in the sample. These results indicate that thermal heating has no effect on the decomposition of ZnFe_2_O_4_ in the absence of a reducing agent.

Under reducing conditions, the decomposition of the ZnFe_2_O_4_ begins at 925°C [18], (figure 4*b*). The TG curve shows a mass loss of about 50% in the temperature range from 823°C to 1165°C. This mass loss is due to the complete evaporation of zinc from ZnFe_2_O_4_ and a reduction of iron oxide to metallic iron. Figure 5*d* indicates that ZnFe_2_O_4_ is completely reduced to metallic iron.

The DSC curve shows a series of reaction peaks at 925°C, 1040°C, 1093°C and 1159°C; these reactions are due to the decomposition of ZnFe_2_O_4_ to zinc and metallic iron. The overall carbothermal reduction of ZnFe_2_O_4_ is considered to be

5.4 $$\text{ZnF}{\text{e}}_{2}{{\text{O}}_{4}}_{\left(s\right)}+4{\text{C}}_{\left(s\right)}={\text{Zn}}_{\left(g\right)}+2{\text{Fe}}_{\left(s\right)}+4{\text{CO}}_{\left(g\right)}$$

First, ZnFe_2_O_4_ decomposes to ZnO and Fe_2_O_3_/Fe_3_O_4_ at 925°C; this result is also reported by Lee *et al*. [20]. After that, the reduction of ZnO and Fe_2_O_3_/Fe_3_O_4_ takes place. The peak at 1040°C is attributed to the reduction of ZnO to elemental zinc vapour [18]. The consecutive peaks at 1040°C, 1093°C and 1159°C represent the transformation of Fe_2_O_3_ to metallic iron according to the reaction: Fe_2_O_3 _→ Fe_3_O_4 _→ FeO → Fe [20].

The mechanism for the overall reaction (5.4) can be written as follows:

5.5 $$\begin{array}{rl} & {\text{ZnFe}}_{2}{{\text{O}}_{4}}_{\left(s\right)}+{\text{C}}_{\left(s\right)}={\mathrm{ZnO}}_{\left(s\right)}+{\text{Fe}}_{2}{{\text{O}}_{3}}_{\left(s\right)} \end{array}$$

5.6 $$\begin{array}{rl} & {\text{C}}_{\left(s\right)}+{\text{O}}_{\left(g\right)}={\text{CO}}_{\left(g\right)}, \end{array}$$

5.7 $$\begin{array}{rl} & {\text{ZnO}}_{\left(s\right)}+{\text{CO}}_{\left(g\right)}={\text{Zn}}_{\left(g\right)}+{\text{CO}}_{2(g)}, \end{array}$$

5.8 $$\begin{array}{rl} & 3{\mathrm{Fe}}_{2}{\text{O}}_{3(s)}+{\text{CO}}_{\left(g\right)}=2{\mathrm{Fe}}_{3}{\text{O}}_{4(s)}+{\text{CO}}_{2(g)}, \end{array}$$

5.9 $$\begin{array}{rl} & {\text{Fe}}_{3}{\text{O}}_{4(s)}+{\text{CO}}_{\left(g\right)}=3{\mathrm{FeO}}_{\left(s\right)}+{\mathrm{CO}}_{2(g)}, \end{array}$$

5.10 $$\begin{array}{rl} & {\text{FeO}}_{\left(s\right)}+{\text{CO}}_{\left(g\right)}={\mathrm{Fe}}_{\left(s\right)}+{\text{CO}}_{2(g)} \end{array}$$

5.11 $$\begin{array}{rl} \text{and}\;\; & {\text{C}}_{\left(s\right)}+{\mathrm{CO}}_{2(g)}=2{\mathrm{CO}}_{\left(g\right)}. \end{array}$$

ZnFe_2_O_4_ decomposes to ZnO and Fe_2_O_3_, as in the reaction (5.5), in the range of 925–968°C. A CO/CO_2_ atmosphere is produced by reduction processes and the Boudouard mechanism (reactions (5.6) and (5.11)). Gas/solid reactions between carbon monoxide and metal oxide (ZnO, Fe_2_O_3_, Fe_3_O_4_ or FeO) take place, and the reduction of zinc oxide and iron oxide to zinc and iron metals takes place.

### 5.2. Dielectric properties

For both samples, the dielectric values at room temperature were quite low, therefore both ZnO and ZnFe_2_O_4_ are poor microwave absorbers. The ZnFe_2_O_4_ sample has significantly higher dielectric properties compared to ZnO. The change in the dielectric values ($\varepsilon^{\prime }$ and $\varepsilon^{\prime \prime }$) of a material as a function of temperature is a critical factor that determines the compound efficiency to absorb microwave radiation [15].

#### 5.2.1. Zinc oxide

**Figure 6.**
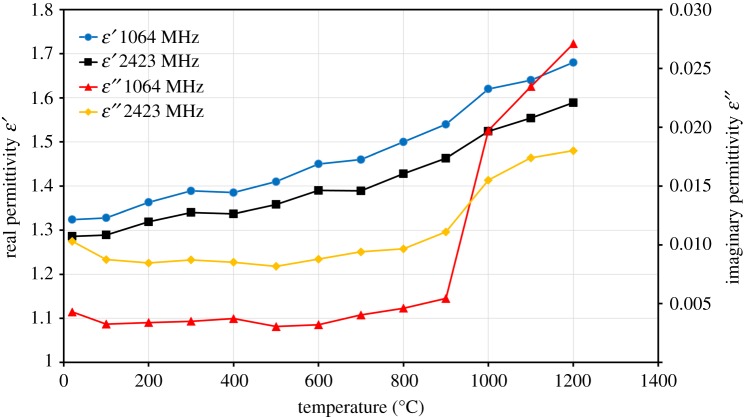
Real ($\varepsilon^{\prime }$) and imaginary ($\varepsilon^{\prime \prime }$) permittivities of ZnO as a function of temperature at frequencies of 1064 MHz and 2423 MHz.

The real ($\varepsilon^{\prime }$) and imaginary ($\varepsilon^{\prime \prime }$) permittivities of ZnO at frequencies of 1064 MHz and 2423 MHz and in the temperature range between 20°C and 1200°C are presented in figure 6. The trends at the two frequencies were similar for both of the permittivities. The changes in real permittivity ($\varepsilon^{\prime }$) of ZnO with temperature are insignificant. Figure 6 shows that $\varepsilon^{\prime }$ increased slightly with an increase in temperature from 20°C to 1200°C (from 1.28 to 1.58) at a frequency of 2423 MHz.

The changes in imaginary permittivity ($\varepsilon^{\prime \prime }$) are larger and the effect of temperature is more significant at higher temperatures. The $\varepsilon^{\prime \prime }$ decreases slightly with an increase in temperature (100°C) due to removal of free water, and then stays approximately constant until a temperature of 800°C. When the temperature exceeds 900°C, a sharp rise of $\varepsilon^{\prime \prime }$ from 0.0051 to 0.027 at 1064 MHz frequency is observed. The increase in $\varepsilon^{\prime \prime }$ at high temperature is attributed to the increase in the electrical conductivity. TG–DSC curves of ZnO did not confirm any phase change in the whole temperature range (figure 3*a*). From the comparison of the DSC curve, the greater changes in imaginary permittivity ($\varepsilon^{\prime \prime }$) appear at temperatures when the sample begins sintering.

**Figure 7.**
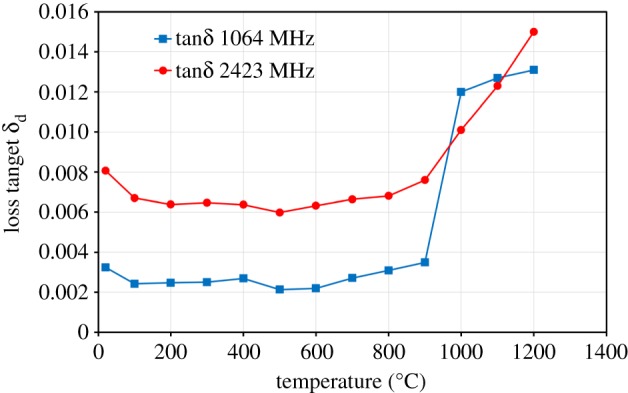
Loss tangent (*δ*_d_) of ZnO as a function of temperature at frequencies of 1064 MHz and 2423 MHz.

The ratio of imaginary ($\varepsilon^{\prime \prime }$) to real ($\varepsilon^{\prime }$) permittivities is called the loss tangent ($\varepsilon^{\prime \prime }$/$\varepsilon^{\prime }$). When the loss tangent is above 0.05, the material is considered to heat well under microwave irradiation [21]. Figure 7 shows the loss tangent of ZnO as a function of temperature at frequencies of 1064 MHz and 2423 MHz. It can be seen that the value remains constant in the temperature range of 100–900°C; above 900°C, the loss tangent increases considerably with further increase in temperature. The maximum loss tangent value for ZnO is below 0.015, which indicated that this material does not heat well under microwave irradiation.

#### 5.2.2. Zinc ferrite

**Figure 8.**
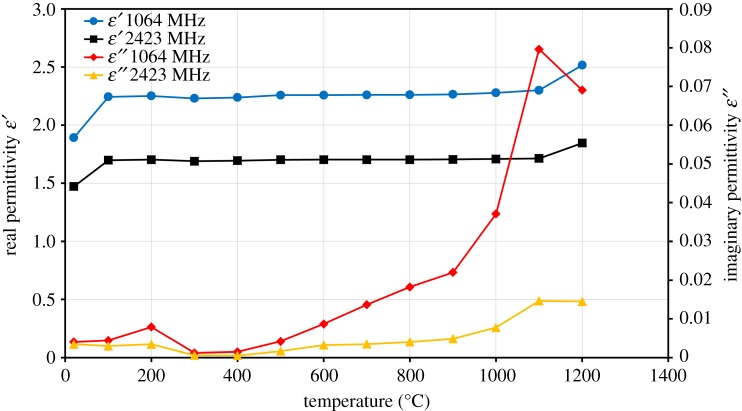
Real ($\varepsilon^{\prime }$) and imaginary ($\varepsilon^{\prime \prime }$) permittivities of ZnFe_2_O_4_ as a function of temperature at frequencies of 1064 MHz and 2423 MHz.

The real ($\varepsilon^{\prime }$) and imaginary ($\varepsilon^{\prime \prime }$) permittivities of ZnFe_2_O_4_ at frequencies of 1064 MHz and 2423 MHz and in the temperature range between 20°C and 1200°C are presented in figure 8. The real ($\varepsilon^{\prime }$) permittivity of ZnFe_2_O_4_ increases slightly with an increase in temperature to 100°C, and then stays almost constant (between 1.7 and 1.71) over the temperature range from 100°C to 1100°C at 2423 MHz frequency. There is a slight increase towards the high end of the temperature range.

The imaginary ($\varepsilon^{\prime \prime }$) permittivity of ZnFe_2_O_4_ decreases slightly with an increase in the temperature (300°C) due to removal of free water. The TG curve shows a mass loss of about 2% in this temperature range (figure 4*a*); this mass loss is attributed to the evaporation of physically adsorbed water. After that, there is a small increase in the $\varepsilon^{\prime \prime }$ with an increase in temperature until 900°C, and then it rapidly increases when the temperature exceeds 900°C. The rise of $\varepsilon^{\prime \prime }$ from 0.02 to 0.08 at 1064 MHz frequency is observed. The changes in imaginary ($\varepsilon^{\prime \prime }$) permittivity are associated with the sintering of the sample at high temperatures (see DSC curve in figure 4*a*.

**Figure 9.**
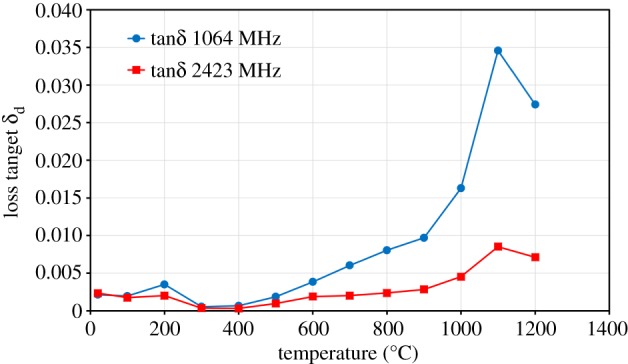
Loss tangent (*δ*_d_) of ZnFe_2_O_4_ as a function of temperature at frequencies of 1064 MHz and 2423 MHz.

The loss tangent for ZnFe_2_O_4_ as a function of temperature at frequencies of 1064 MHz and 2423 MHz is shown in figure 9. There is a small increase in the loss tangent of ZnFe_2_O_4_ in the temperature range of 100–800°C, and a significant increase takes place when the temperature exceeds 900°C (figure 9). The maximum loss tangent value for ZnFe_2_O_4_ is below 0.035, which means that ZnFe_2_O_4_ does not heat well under microwave irradiation. The behaviour of the loss tangent for both ZnO and ZnFe_2_O_4_ with temperature coincides with the behaviour of the imaginary ($\varepsilon^{\prime \prime }$) permittivity.

### 5.3. Heating rate with microwaves

The microwave heating rates of both ZnO and ZnFe_2_O_4_ are very slow due to their low dielectric values as mentioned in the previous section. This result is in agreement with other works [11]. In the experiments, in order to decrease the heat loss from the surface of the samples and to ensure efficient heating, the crucibles were insulated by alumina block.

#### 5.3.1. Zinc oxide

**Figure 10.**
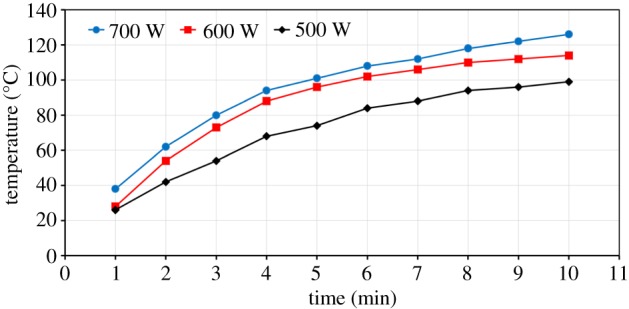
Effect of the microwave power level on the heating rate of ZnO.

The heating behaviour of ZnO at three different power levels of 500, 600 and 700 W is presented in figure 10. ZnO appears to be a poor microwave absorber and its temperature does not exceed 120°C. With increase in power intensity, the measured sample temperature is observed to slightly increase. For example, the sample exposed to 700 W of microwave power at an exposure time of 10 min attained a temperature of 126°C, while a sample exposed to 500 W of microwave power for the same exposure time attained a temperature of 99°C (figure 10). The effect of exposure time on sample temperature is shown in figure 10. At a microwave power of 700 W, the temperature at 4 min was 94°C, and then increased slowly to reach about 126°C after 10 min.

**Figure 11.**
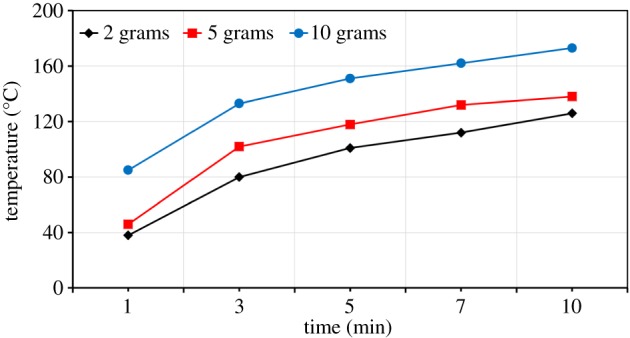
Effect of the sample mass on the temperature of ZnO.

The effect of sample mass on the sample temperature at 700 W is shown in figure 11. For ZnO, at a sample mass of 2 g, the temperature at 10 min was about 126°C. The sample temperature increased to about 138°C when the mass was increased to 5 g, and 173°C was achieved for a 10 g sample (figure 11). The increase in sample temperatures with increase in sample mass was also investigated by other researchers [22,23]. These results can be explained for the same cross-sectional area of the crucible; as the sample mass is increased, there is a reduction in the surface area to volume ratio and this reduces the heat loss from the interior, leading to a higher bulk sample temperature [22,23].

#### 5.3.2. Zinc ferrite

**Figure 12.**
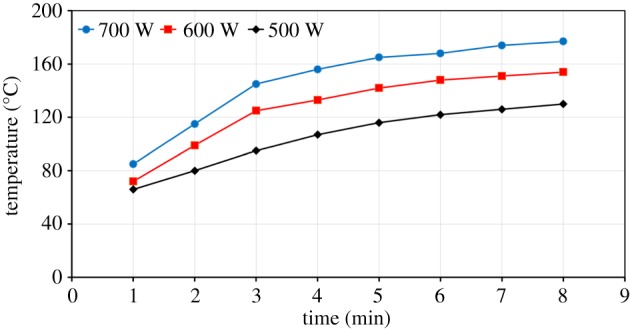
Effect of the microwave power level on the heating rate of ZnFe_2_O_4_.

The heating behaviour of ZnFe_2_O_4_ at three different power levels of 500, 600 and 700 W is presented in figure 12. The results show that the microwave heating of ZnFe_2_O_4_ is faster in comparison to that of ZnO, but is still a slow heating rate. Increasing the microwave power from 500 to 700 W resulted in an increase in sample temperature from 130°C to 177°C after 8 min of heating time. Figure 12 shows that the measured sample temperature increases with increase in exposure time. At a microwave power of 700 W, the sample temperature rose sharply to about 150°C within 3 min. Then, the sample temperature increased gradually and reached 177°C after 8 min (figure 12).

**Figure 13.**
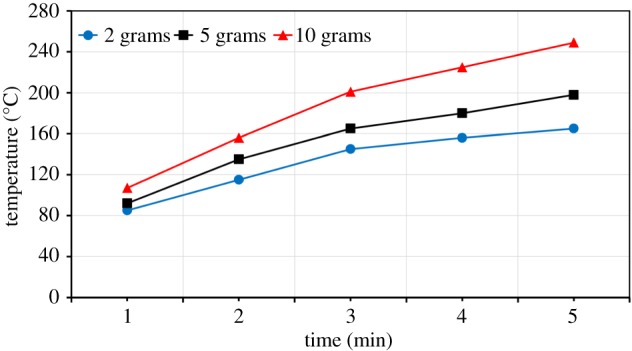
Effect of the sample mass on the temperature of ZnFe_2_O_4_.

The effect of the sample mass on the temperature of ZnFe_2_O_4_ at 700 W is shown in figure 13. At 700 W and 5 min of exposure time, the 2 g sample attained a temperature of about 165°C. At 10 g of sample, the temperature rose to 249°C (figure 13).

The results show that the sample temperature increased with increase in microwave power and sample mass. The low heating rates of these materials are in accordance with the measured values of the dielectric properties of both materials (figures 6–9).

**Figure 14.**
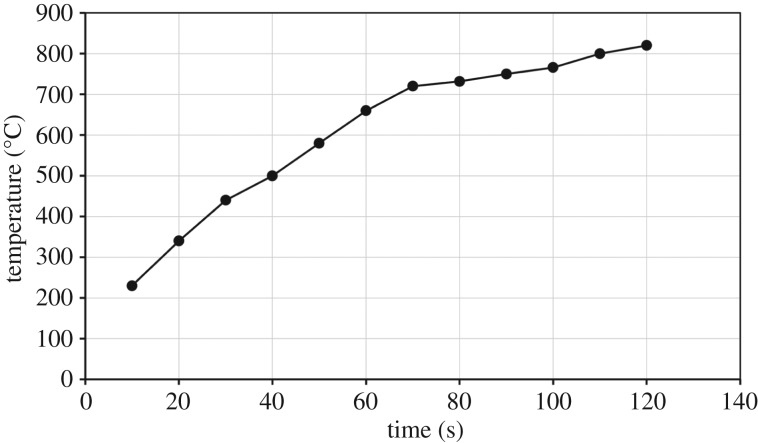
Microwave heating of graphite at a microwave power level of 700 W.

In this study, graphite was used as a reducing agent and absorbent of microwave energy. From the achieved results, both ZnO and ZnFe_2_O_4_ do not effectively absorb microwave energy, whereas graphite absorbs microwave energy very well. The results show that graphite heats easily up to about 800°C in less than 2 min (figure 14). So graphite will play an important role in heating of ZnO and ZnFe_2_O_4_/graphite mixtures.

### 5.4. Carbothermic reduction

In the reduction experiments, a higher microwave power of 700 W was used. The effects of the stoichiometric amount of the reducing agent on the reduction degree of ZnO and ZnFe_2_O_4_ were studied.

#### 5.4.1. Zinc oxide

**Figure 15.**
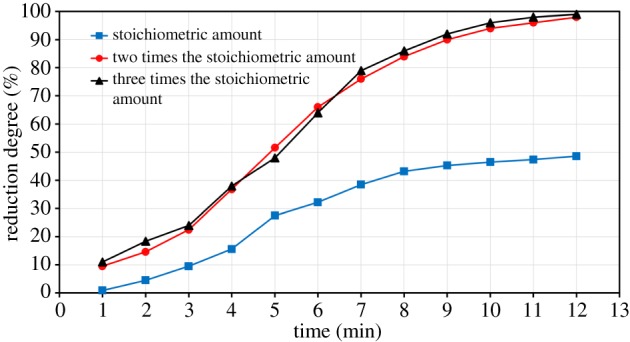
Effect of the stoichiometric amount of graphite on ZnO reduction degree (700 W microwave power).

The reduction degrees of ZnO with one, two and three times the stoichiometric amount of graphite (according to equation (4.2)) at a microwave power of 700 W are presented in figure 15. It is apparent that the reduction degree is significantly affected by the amount of the reducing agent added.

Figure 15 shows the effect of the amount of reducing agent on the reduction degree of ZnO. With the stoichiometric amount of graphite, the reduction of ZnO does not exceed 48%. The complete reduction of ZnO is not achieved because the amount of carbon added is insufficient. To achieve the complete reduction of ZnO, the amount of reducing agent was increased to two times the stoichiometric amount. Almost a complete reduction degree (99%) has been achieved at 12 min using 700 W microwave power. The addition of excess carbon tends to increase the amount of CO formed by the Boudouard reaction, and thus accelerates the ZnO reduction rate [15]. The addition of an excess amount of carbon to the ZnO has an insignificant effect on the reduction rate of ZnO. Figure 15 showed that increasing the amount of the reducing agent to three times the stoichiometric amount has the same reduction degree of two times the stoichiometric amount. Therefore, two times the stoichiometric amount of graphite is sufficient to achieve the complete reduction of ZnO.

#### 5.4.2. Zinc ferrite

**Figure 16.**
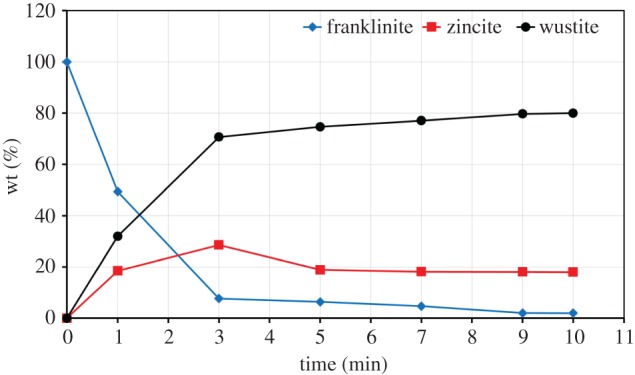
Decomposition of zinc ferrite as a function of time (one stoichiometric amount of graphite, 700 W microwave power).

The rates of ZnFe_2_O_4_ decomposition were interpreted based on the results of the XRD. The concentrations of ZnFe_2_O_4_, ZnO, FeO and metallic iron phases were plotted against the processing time. Figure 16 presents the results of reduction of ZnFe_2_O_4_ with one stoichiometric amount of graphite. Initially, ZnFe_2_O_4_ decomposes to the ZnFe_2_O_4_, ZnO and FeO phases after 1 min, but grains containing ZnFe_2_O_4_ are still present. Figure 16 shows that the franklinite phase decreases with increase in reaction time, but the complete decomposition of ZnFe_2_O_4_ is not attained and traces of franklinite are detected in the sample after 10 min. The wustite content increases with increase in reaction time from 0 to 5 min, but ZnO content decreases from 3 to 5 min. When the reaction time reaches 5 min, no additional reduction of ZnO occurs and the wustite content remains unchanged. At 9 min, no additional reactions occur in the sample. Figure 16 indicates that ZnO and ZnFe_2_O_4_ phases are still present; this is due to the fact that the amount of carbon is insufficient for the complete decomposition of ZnFe_2_O_4_ and removal of the Zn from the sample.

The decomposition of ZnFe_2_O_4_ was also reported by other researchers [24,25]. Nyirenda [24] reported the decomposition of ZnFe_2_O_4_ at 750°C using carbon monoxide as the reducing agent. Hua *et al*. [25] reported the formation of a mixture of Fe_3_O_4_, FeO and zinc vapour at 1000°C. Li *et al.* [26] indicated that ZnFe_2_O_4_ roasted at 750°C for 1 h decomposed into ZnO and Fe_3_O_4_. The results indicated that the rate of ZnFe_2_O_4_ decomposition with microwave heating is faster than with conventional heating. The formation of a mixture of FeO and ZnO with microwave instead of Fe_2_O_3_/Fe_3_O_4_ and ZnO with conventional heating is due to fast microwave reaction and reduction of Fe_3_O_4_ into FeO in a short time.

**Figure 17.**
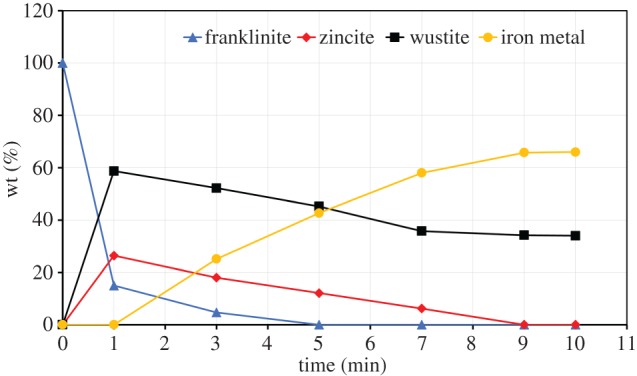
Decomposition of zinc ferrite as a function of time (two times the stoichiometric amount of graphite, 700 W microwave power).

At two times stoichiometric amount of graphite, ZnFe_2_O_4_ begins to decompose at 1 min, and the decomposition degree increases with increase in reaction time. A further increase in reaction time (at 5 min) resulted in ZnFe_2_O_4_ being consumed with the formation of FeO, metallic iron and ZnO. Figure 17 indicates that it is easy for ZnFe_2_O_4_ to completely transformed into ZnO and wustite but difficult for reaction under one stoichiometric amount of carbon. The transformation of wustite into metallic iron is promoted as the concentration of FeO decreased compared to the metallic iron content (figure 17). It should be noted that the disappearance of ZnO indicated that ZnO is reduced to metallic zinc and is completely removed at 9 min of reaction time. After 9 min, the concentrations of phases become constant, which means that the reaction is completed.

**Figure 18.**
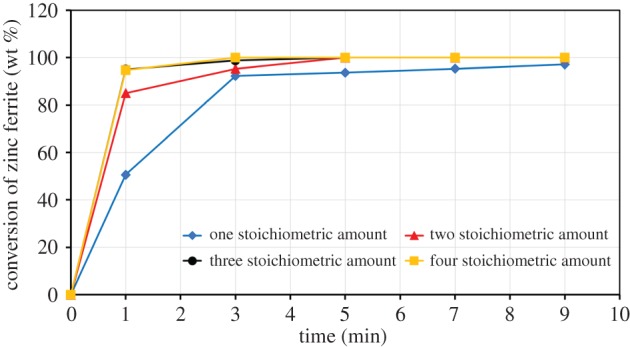
Rate of zinc ferrite conversion against time with different stoichiometric amounts of graphite.

Figure 18 shows the conversion degree of ZnFe_2_O_4_ at different stoichiometric amounts of carbon; the results indicate that the decomposition degree has increased with increase in carbon content from one to three times of the stoichiometric value at constant time. At 1 min, in the cases of one, two and three times the stoichiometric amount of carbon, the decomposition degrees of franklinite are 50.4%, 84.6% and 94.7%, respectively. At 5 min, ZnFe_2_O_4_ is completely decomposed when the stoichiometric amount of carbon is more than one.

**Figure 19.**
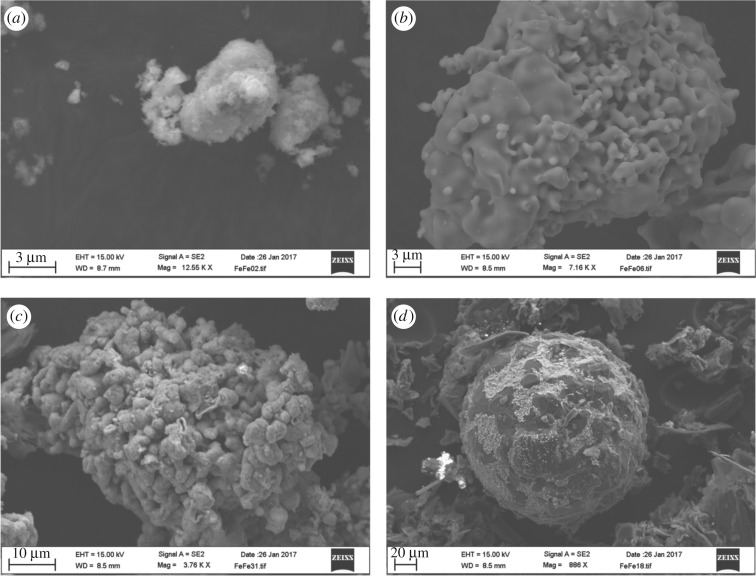
SEM images of zinc ferrite after reduction with two times the stoichiometric amount of graphite at different times. (*a*) Unreacted; (*b*) at 1 min; (*c*) at 3 min; (*d*) at 9 min.

The morphology changes of ZnFe_2_O_4_ after reduction at different times were examined by SEM–EDS, and the results are presented in figure 19. SEM micrographs of unreacted ZnFe_2_O_4_ and after 1 min of reaction time using a microwave power of 700 W are shown in figure 19*a,b*. Figure 19*a* reveals that ZnFe_2_O_4_ is a dense and sphere-like grain. Figure 19*b* shows that many small pores are generated on the surface of the franklinite grain after 1 min of treatment. These pores are generated due to the evaporation of Zn from the franklinite grain (figure 19*b*). These results were also observed by Lee *et al*. [20]. Figure 19*c* shows the wustite grain formed after 3 min of microwave heating. Spherical metallic iron is observed at the final stage of the reaction (figure 19*d*).

**Figure 20.**
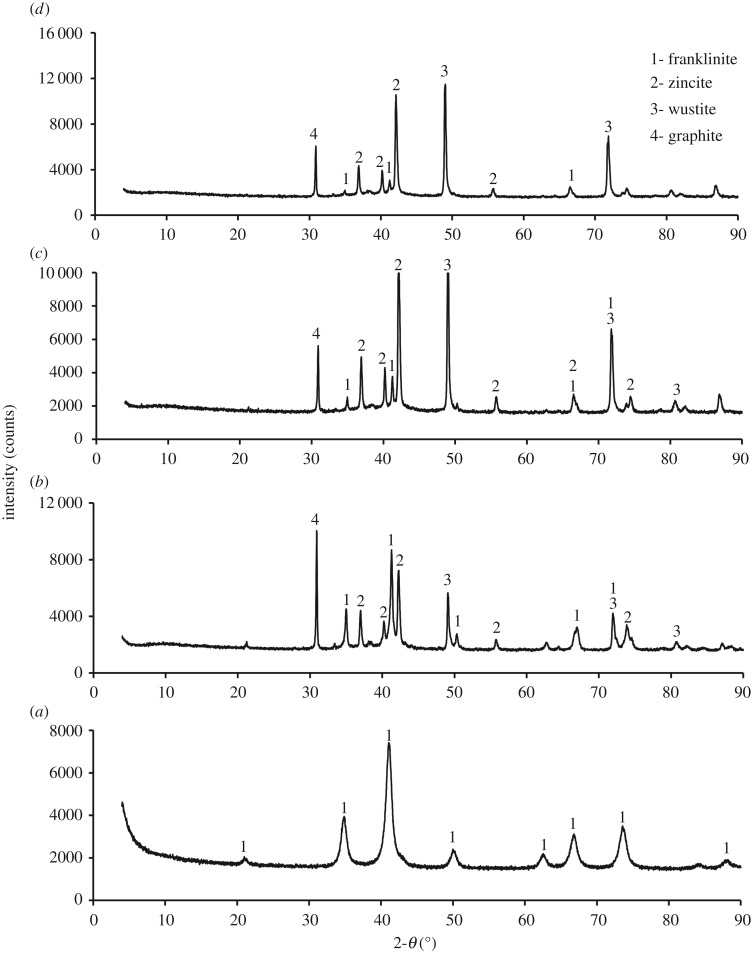
XRD patterns for zinc ferrite reduced with one stoichiometric amount of graphite. (*a*) Unreacted; (*b*) at 1 min; (*c*) at 3 min; (*d*) at 9 min.

The XRD patterns of the ZnFe_2_O_4_ reduced with one stoichiometric amount of graphite at different reaction times are shown in figure 20. The main phases of the reduced sample are franklinite, ZnO and wustite. A significant decrease in the intensity of the franklinite peak is observed at 9 min (figure 20*d*), while the wustite peak intensity increases with increase in reaction time.

## 6. Conclusion

Dielectric properties and carbothermic reduction of ZnO and ZnFe_2_O_4_ by microwave heating have been studied. The following conclusions can be drawn:

(i) The dielectric values of ZnO and ZnFe_2_O_4_ at room temperature were quite low. The influence of temperature on the real ($\varepsilon^{\prime }$) and imaginary ($\varepsilon^{\prime \prime }$) permittivities indicated that temperature has a more significant effect on the imaginary permittivity than on the real permittivity, and the effect of temperature is more significant at higher temperatures.
(ii) The poor microwave absorption of ZnO and ZnFe_2_O_4_ is due to the low dielectric values of both samples. The microwave heating rate showed that the sample temperature increased with increase in microwave power and sample mass.
(iii) The reduction degree of ZnO is significantly affected by the amount of the reducing agent; with two times the stoichiometric amount of carbon, almost a reduction degree of 99% has been achieved at 12 min using 700 W microwave power.
(iv) At two times the stoichiometric amount of graphite, ZnFe_2_O_4_ completely transformed into ZnO and wustite in 3 min. The phase composition of ZnFe_2_O_4_ indicated that the ZnFe_2_O_4_ decomposed in three stages: reduction of ZnFe_2_O_4_ to ZnO and FeO, reduction of ZnO to Zn vapour and reduction of FeO to Fe.

## Data accessibility

All the data used for this study are found in the manuscript.
